# Athletes' and spectators' expenditures during a medium-sized sport event: the case study of the Italian National University Sport Championship

**DOI:** 10.3389/fspor.2025.1528503

**Published:** 2025-08-29

**Authors:** Luca Rossini, Lavinia Falese, Alexandro Andrade, Arthur Dutra, Marco Giglio, Daniela Federici

**Affiliations:** ^1^ Department of Economics and Law, University of Cassino and Southern Lazio, Cassino, Italy; ^2^ European University of Technology EUt+, European Union; ^3^ Department of Human Sciences, Society and Health, University of Cassino and Southern Lazio, Cassino, Italy; ^4^ Laboratory of Sport and Exercise Psychology, Health and Sports Science Centre, Department of Physical Education, Santa Catarina State University/UDESC, Florianópolis, Brazil

**Keywords:** sport events, economic impact, University sport, participants expenditures, event tourism, spending patterns

## Abstract

**Introduction:**

The present study investigates the economic impact of the 2022 Italian National University Sport Championship (CNU), held in Cassino, Italy, with particular emphasis on the spending patterns of athletes, technical staff, and spectators.

**Methods:**

A cross-sectional survey was employed during the event to collect data from 963 participants, separated into two different groups: athletes/staff (Group A), and spectators (Group B). The survey addressed participants' spending patterns, satisfaction levels, and event experiences. The DEC (Direct Economic Impact Calculator) tool was employed to estimate the total economic contributions, while further analyses explored the differences in spending according to participant satisfaction and attributes.

**Results:**

The total direct economic impact of the event was estimated at €1,323,572, including €50,000 in organizer expenditures, which contributed to the support of 22 local jobs. Non-local spectators had a notable economic impact, with an average daily expenditure of €223, compared to €124 by athletes and staff. Satisfaction with public transport, the possibility to purchase local products, and positive interactions with the staff working at the event are among the key factors positively influencing spending. In contrast, engagement in ceremonial activities and feelings of nervousness were associated with lower spending levels.

**Discussion:**

These results underscore the significant economic influence of medium-sized sports events on local economies, highlighting the importance of improving visitor experiences to maximize economic benefits. The findings offer significant implications for event organizers and policymakers who seek to enhance the economic and social outcomes associated with these events.

## 1 Introduction

Sports events have a variety of effects, which are categorized as immediate event impacts and long-term legacies. Despite having different meanings, the two terms are frequently and mistakenly used interchangeably. Event impacts are immediate changes in the local economy, culture, or environment, often noticed during and shortly after the event. On the contrary, legacies frequently refer to longer lasting benefits that persist even after the event's conclusion (1).

The immediate economic repercussions of sports events typically include enhanced revenue for local businesses and a boost in tourism and can materialize even before the event begins, especially if significant investments are required for the infrastructure development (2). Organizing and hosting sports events, for instance, can lead to an increase in visitor spending on food, accommodation, transportation and several other services, consequently benefitting local businesses and the host city through additional tax revenues. The favorable economic effects, with the promotion of the sport itself, are crucial for substantiating the investments in hosting these events (3). Nevertheless, the economic consequences of sporting events are not always advantageous. Increased prices for local products and services, congestion, and the expenses associated with infrastructure development are just some of the potential adverse effects. Additionally, if the anticipated visitor numbers do not materialize, the host city may face financial losses. Consequently, it is necessary to rigorously evaluate and regulate these aspects to optimize benefits and reduce adverse effects (4).

Despite the importance of comprehending these economic effects and their assessment, most of the research has focused on large-scale events, resulting in a gap in the literature about small and medium-sized events (5, 6) and ignoring the special dynamics and contributions of competitions at smaller scales (7). In order to better contextualize these events, Gratton, Dobson, and Shibli propose a classification of major sports events by their sporting outcomes (e.g., national, European, or World Championships) rather than their economic importance, specifying that not all events that are major in sporting terms are also impactful in economic terms (8). This classification offers a useful framework for assessing the scope, potential impact, and strategic value of events beyond the mega-event paradigm. While types A and B events refer to global international, spectator-driven events with high media exposure, types C and D include more modest, often competitor-driven events such as junior championships, sub-elite events or national competitions which tend to have lower commercial impact, produce lower daily expenditures but remain important for local development (9). This categorization emphasizes the importance of investigating type C and D events, which have not received enough attention in the academic literature despite their prevalence. Although mega-events like the Olympics and the World Cup receive predominant scholarly and media focus, smaller-scale events offer substantial prospects for community engagement, local economic advancement, and the encouragement of sports participation (10–12). These events, although modest in scale, are instrumental in shaping the sporting environment and developing a sense of community among participants and spectators (12) and may even be held more frequently due to their relatively low investment demand, especially when compared to mega-events.

The economic benefits produced by an event can be measured through several methods, such as cost-benefit analysis, income-outcome assessments, or individual expenditure assessments. For small and medium-sized sports events the primary approach is the direct incremental spending, which emphasizes the supplementary expenditures produced by the event (13). Other methods include input-output (I-O) analysis, and computable general equilibrium (CGE) models, which account for price changes and supply constraints, thereby being considered more suitable for long-term impacts (14). Cost-benefit analysis (CBA) is comprehensive, but data-intensive (15). For smaller events, qualitative assessments and CGE modeling are rare, while I-O analysis remains the preferred method due to its regional applicability, despite having this strategy faced criticism for its assumptions and potential overestimation of economic impacts (14, 16).

Precise economic impact evaluations necessitate the acquisition of primary data regarding visitor spending (17, 18), typically obtained through direct surveys at events to mitigate recall bias and ensure accurate spending estimates (19, 20). Research highlights the significance of non-resident expenditure, which introduces fresh capital into the local economy, whereas local expenditure is seen as recirculation (21, 22). Factors affecting spending encompass visitor satisfaction, perceived quality, duration of stay, accommodation type, income levels, and the nature of the event and sport (23). Non-resident participants and spectators significantly contribute to the local economy, especially through expenditures on accommodation (24). Spending patterns differ across event categories and competitive levels, with prolonged tournaments and greater competitive levels resulting in increased direct spending (25). Market segmentation factors and individual factors such as wealth, age, and previous interest in the sport or event might also affect spending patterns. Previous research has emphasized the need to differentiate among market segments when estimating economic impacts of sporting events. Solberg et al. showed that groups such as media representatives, sponsors, business travelers, and athletes exhibit distinct spending patterns, often driven by their roles and funding sources (26). Attendees with more discretionary incomes or fervent enthusiasm are more inclined to expend additional resources (27).

General literature on event tourism, not specific for sport events, found that expenditures are influenced by a variety of factors that encompass both intrinsic and extrinsic variables. A key component is the perceived quality, which involves a variety of factors, including the overall experience, facilities, and organization. Higher quality events usually result in higher levels of satisfaction, which in turn increases the probability of spending (28). Memorable experiences also significantly influence spending behaviors. Events that provide distinctive, engaging, and emotionally impactful experiences frequently lead to increased expenditures from participants desiring to enhance their enjoyment and acquire souvenirs (29). Satisfaction serves as a vital element, functioning as a mediator between perceived quality and expenditures. Increased enjoyment frequently results in increased expenditure, since satisfied attendees are more prone to extend their visit, acquire products, and participate in additional activities associated with the event (30). External factors, such as the economic conditions of the host city, the availability of supplementary services (for instance, food and accommodation), and marketing initiatives, all influence spending. Effective marketing strategies can increase the perceived value of the event, consequently attracting more attendees and encouraging a greater amount of spending (31). Eventually, social factors, such as the presence of family and friends, can affect spending patterns, since group dynamics frequently contribute to higher expenditures on communal activities and shared experiences (32).

Despite existing studies on non-sport event tourism and mega sport events, there is a gap in the literature regarding the determinants of economic impact for medium and small-sized sports events (33, 34), especially the effect of experiential factors such as satisfaction and perceived quality.

Through detailed analysis, this study aims to investigate not only the economic impact but also the experiential and psychosocial dimensions that drive expenditures in medium-sized, athlete-driven sports events and offer practical recommendations for enhancing the quality and appeal of these events. While some of the methods and tools to investigate direct economic impacts and expenditures have been widely applied in the context of sport events, in our study their implementation has been paired with the analysis of emotional, satisfaction-based, and behavioral determinants of spending, expanding their application beyond standard descriptive use and offering novel insights into how visitor experience correlates with local economic benefit.

This study seeks to address three main research questions: what is the direct economic impact of a medium-sized, university-level sporting event on the host economy? How do variables such as individual satisfaction, perception of event quality, event expectations, sport performance, memorable experiences, participation in collateral activities, relate to individual expenditure during the event? And do spending patterns differ between distinct participants segments?

Building on previous research in sport tourism and event studies, we hypothesize that higher satisfaction, stronger positive emotional engagement, and positive behavioral intentions (such as the intention to return or recommend the event) are positively associated with individual spending. We also expect that spectators will report higher spending than athletes, and that non-local visitors will spend more than local attendees.

Studies such as this can contribute to ongoing local development and community building, providing valuable insights for event organizers, policymakers, and community stakeholders to optimize benefits.

## 2 Methodology

This study focuses on the economic impact and individual expenditures of athletes and spectators in the Italian National University Sport Championship (CNU) held in Cassino, Italy, in May 2022, an annual event attracting around 2500 university athletes from all over Italy, competing in numerous sports. The research was approved by the Institutional Review Board of the University of Cassino and Southern Lazio (protocol n.7924).

### 2.1 Data collection

The study is cross-sectional and the data were collected during the last day of the Event (the date changes according to the sport since not all the sports finished the same day).

For data collected from individuals, no a-priori statistical calculation of sample size was performed but, since the Event is multi-sport and gender, the data collection was programmed and developed in order to try to ensure the representativeness of sport discipline/gender.

In order to allow for a specific assessment of net additional income generated within the local area, the host economy for this study is defined as the municipality of Cassino (Italy) and its immediate economic surroundings. This includes businesses and services that directly benefitted from event-related visitor expenditure, such as accommodation, restaurants, retail, and local transportation.

### 2.2 Sample

People who took part in the investigation were divided into two different groups (group A, group B). Group A consisted of athletes and technical staff while group B included the spectators.

No a-priori sample size calculation was performed; however *post hoc* power analysis was conducted to evaluate the adequacy of the sample size collected and efforts were made to ensure representativeness. In Group A, a sample of 742 participants was obtained from an estimated total population of 2,310 persons, whereas Group B had 221 questionnaires gathered from an estimated population of 300 spectators. For Group A, we used participant lists provided by the organizer (including sport, gender, university, and city) to guide proportional sampling across sports. For Group B, data collection was conducted throughout the event, targeting a broad mix of spectators.

The power of the study was then calculated using a medium effect size (*d* = 0.5) and a significance level (α) of 0.05. The analysis indicated a power of 1.0 (100%), suggesting that the sample size was more than sufficient to detect a statistically significant effect, if present. This high-power level demonstrates confidence in the study's ability to detect medium or larger effects within the population.

### 2.3 Instruments

#### 2.3.1 Economic impact calculator

The Direct Economic Impact Calculator (DEC), provided by https://www.EventImpacts.com, has been utilized to assess the direct economic impact. This tool is recognized for its methodological clarity and has been widely adopted especially in the UK to evaluate the direct economic benefits of sporting and cultural events. This approach is recognized as a very reliable instrument for evaluating the economic impacts of events (1).

The DEC estimates the amount of net additional expenditure generated within a defined host economy as a direct consequence of staging the event and it incorporates visitor surveys to capture spending that is directly attributable to event attendance and organizer spending within the local economy. The process involves two core components: collecting and averaging spending data from a sample of event visitors and scaling it up to represent the total visitor population and assessing the organizers' net local expenditure, calculated as spending with local suppliers minus revenue generated within the host economy. For accurate estimation, this process requires a clearly defined geographic scope, reliable visitor counts, and robust survey samples. Additionally, calculations consider potential deadweight (spending that would have occurred anyway) and leakages (spending that exits the local economy), which are used to isolate the truly additional economic impact. Results are presented in terms of direct economic impact, spectators' spending, attendees' spending, direct leakages and job supported generated by the event.

#### 2.3.2 Questionnaires

To collect data from athletes, staff and spectators, useful for the Direct Economic Impact assessment and for the detailed statistical analysis, we used a questionnaire that was uploaded on Google Form platform. The link was sent out through university sport centers’ mailing lists, social network and shared by volunteers during the event. A paper-pencil version of the questionnaire was also realized in order to facilitate the data collection on site. All the individuals interested in participating in the research were invited to read the detailed information on the study's aims and the statement on anonymous answers' mode. The informed consent and the authorization to process sensitive data will be also requested in accordance with the Italian law (196/2003 and subsequent amendments and additions regarding personal data). The investigation was carried out following the rules of the Declaration of Helsinki from 1964, and then revised in 2000. Two different versions of self-administered questionnaires were used to collect the data according to the person filling in the survey.

The questionnaires included adapted items from validated scales measuring satisfaction, emotional engagement, and behavioral intention. While we used the full scale for certain variables, only the most relevant items were selected from other validated scales to ensure contextual appropriateness for the event type and participant demographics. Items relating to service quality, brand loyalty, or commercial spending were excluded, as they did not apply uniformly to our sample. To assess the internal consistency of the adapted multi-item scales, we calculated Cronbach's alpha for those multi-item constructs that were conceptually coherent and had a sufficient number of items to justify scale-level reliability testing and for both samples (athletes/staff and spectators). For Questionnaire A (athletes/staff), Cronbach's alpha was 0.85 for Expectations, 0.94 for Quality Perception, and 0.87 for Memorable Tourism Experience. For Questionnaire B (spectators), alpha values were 0.89, 0.93, and 0.92 respectively for the same constructs. Results showed strong reliability across all constructs indicating that the adapted scales have excellent internal consistency across both respondent groups.

##### 2.3.2.1 Group A: questionnaire for athletes and technical staff

Section 1: socio-demographic data (gender, age, type of sport, area of study, family's income, etc.); Section 2: information about the stay (days of stay, transportation, accommodation, additional activities/services, etc.); Section 3: economic impact (expenses for transportation, accommodation, food and beverages, entertainment and recreational time, souvenirs, others) (adapted items from Stynes & White) (16); Section 4: expectations about the Event; Section 5: assessment of the quality of the Event, personal experience and satisfaction (adapted items from Ko et al. and MacIntosh & Nicol) (35, 36); selected items from EVENTQUAL scale by Calabuig-Moreno et al. (37) and SPORTSERV scale by Theodorakis and Kambitsis (38); Section 6: destination image and emotions (selected items from destination emotion scale, DES) (39) and Memorable Tourism Experience (MTE) scale (40); Section 7: behavioral intentions/future intentions; Section 8: team/athlete performance during the event (adapted items from Beccarini & Ferrand and Madrigal) (41, 42); Section 9: suggestions for next events (adapted items from MacIntosh & Nicol) (36).

##### 2.3.2.2 Group B: questionnaire for spectators

Adapted questions of the Group A questionnaire, plus the sections below:

Section 10: physical activity self-assessment (shorter version of IPAQ, validated on an Italian sample by Mannocci et al.) (43); Section 11: assessment of future intentions to be more active (adapted items from Malchrowicz -Mośko et al. and Oshimi et al.) (44, 45); Section 12 (only for locals): social impact and perception of the impact on the local community (adapted items from Getz) (1).

In order to reduce the inclusion of deadweight expenditure, the questionnaire specifically asked respondents to report only spending that was directly related to their participation in or attendance at the event meaning expenditures would not have occurred in the host economy otherwise.

### 2.4 Statistical analysis

We used both descriptive and inferential statistical methods to analyze the spending behavior of athletes, staff, and spectators. Descriptive statistics provided an overview of demographic characteristics, spending patterns, and satisfaction levels. For inferential analysis, we conducted bivariate tests to explore relationships between spending and various factors. Correlation matrix and multiple regression analyses were then performed to identify key predictors of spending, using a stepwise approach to retain only significant variables. The regression analysis was guided by a conceptual framework grounded in previous research on sport and event tourism, which suggests that individual expenditures at events is influenced by a combination of experiential, psychological, and demographic factors. The results were assessed based on coefficient values, *p*-values, and R-squared, with statistical significance set at *p* < 0.05. Both software SPSS and STATA were used to perform the analysis.

## 3 Results

The total sample consisted of 963 individuals divided into two groups: 742 athletes and staff, and 221 spectators.

The sample of athletes and staff, consisted of a total of 742 participants, 81.1% athletes, 13.6% staff and 5.3% referees. Of these, the majority identified themselves as male (57.8%). Geographically, the largest proportion of athletes and staff came from the Central Italy (30.9%) followed by North-West (24.2%) and North-East (23.4%). A variety of sports were represented, with volleyball (14.9%), karate (13.9%), and athletics (12.8%) being the most popular (data not shown in the table). Educational field of study/work varied, with the most common fields being engineering or architecture (20.7%) and sport sciences (20.7%) followed by medicine (11.1%) and economics (9.4%). The majority held a high school diploma (53.9%), and 33.5% had a university degree. In terms of household income, 33.1% reported an income between €15,001 and €28,000, while 22.6% earned less than €15,000. Regarding event expenditure, 53% reported no additional expenses, while 33.6% spent between €1 and €50. Moreover, 79.5% of athletes and staff were attending the Italian National University Games for the first time and were visiting the host city/area for the first time (78.8%). Satisfaction levels among athletes and staff were measured through several indicators. The overall satisfaction with the services provided by the organizing committee was rated at an average of 3.43 out of 5, while satisfaction with their stay averaged 3.63 out of 5. Furthermore, participants found the competitions during the event to be exciting, with a mean score of 3.91 out of 5.

The sample of spectators consisted of 221 individuals, 165 of which were non-locals and 56 locals. The gender distribution included 60.5% males. In terms of age, the largest group was between 19 and 30 years old, making up 54.2% of the sample, followed by those aged 51 or older (26.4%). The mean age was 34 years old mostly from Central Italy (68.3%). Sports preferences varied, with futsal matches being the most attended (21.9%), followed by athletics (11.4%) and volleyball (10.5%). Regarding education, 55.1% of respondents held a high school diploma, while 31.7% had a university degree. The largest professional group was employees or workers (23.4%), while 22.9% were students. Household income was diverse, with 33.6% reporting earnings between €15,001 and €28,000, and 26.2% reporting earnings below €15,000. The average daily spending, excluding accommodation, was €20.60. Among the spectators, 74% were attending the Italian National University Games for the first time, and 58.6% were visiting the host city or area for the first time. Descriptive analysis about the satisfaction focused on items such as “overall, the services offered by the organizing committee are adequate” (mean 3.49 out of 5 on a Likert scale), “the competitions during this event are exciting” (mean 4 out of 5 on a Likert scale) and “this event can have a positive impact on the local economy” (mean 3.9 out of 5 of a Likert scale) (data not shown).

The 2022 Italian National University Games event's analysis showed significant economic impacts. Spectators and participants spent considerable amounts on accommodation, food, transportation, and other services. The Direct Economic Impact Calculator tool from https://www.EventImpacts.com estimated the total direct economic impact to be €1,323,572, including organizer spending of €50,000, supporting 22 jobs.

**Figure 1:**
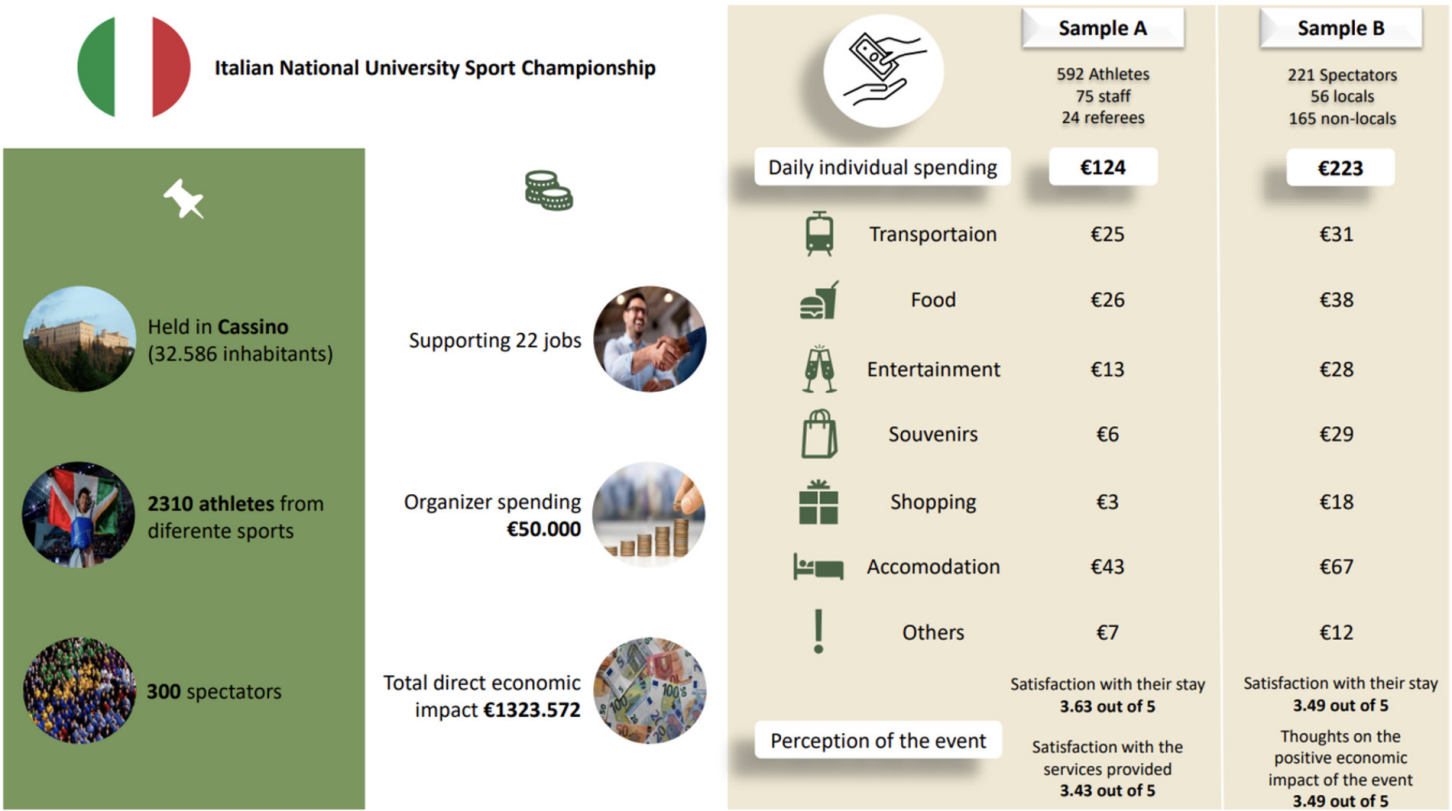
Descriptive characteristics of the event and the economic spending profile of the sample A and B.

Figure 1 presents the main descriptive data of the event, information on the venue and the perception/economic profile of the participants in this study including the expenditures of athletes, staff, and spectators during the event. As shown in Figure 1, the average daily individual spending was markedly different between groups: €124 for athletes and staff (Sample A) and €223 for spectators (Sample B). Spectators spent more in categories such as food (€38), accommodation (€67), and souvenirs (€18), reflecting their role as external consumers compared to athletes whose expenses were often bundled.

For athletes and staff the average daily spending per category was €43 for accommodation, €26 for food, €25 for transportation, €13 for entertainment, €3 for souvenirs, €6 for shopping, and €7 for other expenses.

For spectators the average daily spending was higher, with €67 for accommodation, €38 for food, €31 for transportation, €28 for entertainment, €18 for souvenirs, €29 for shopping, and €12 for other expenses. No significant gender difference in overall expenditures was found.

In order to investigate the relationships between different factors and spending we decided to perform bivariate and regression analysis for the two samples.

We performed several bivariate analyses using the average spending without accommodation as dependent variable. For sample A (athletes and staff), the analysis by age group showed that young adults (19–30 years) and adults (31–50 years) are the age groups with the highest average spending, excluding accommodation costs. Team managers and technical staff spent more on average compared to athletes, and those who had previously participated in the event (in another city) also tended to spend more. A correlation matrix revealed positive, though not always significant, correlations between spending and factors such as curiosity towards new locations/events never seen before (0.33); to visit museums, churches, archaeological sites (0.42); local products tasting (0.38); to visit a location that was on the list of places to visit (0.72). On the other hand, negative correlations were found with: the infrastructures are difficult to reach (−0.04); my team or me is showing great commitment during the competition (−0.06). We also carried out a multiple regression analysis to show the relationship between various items related to the satisfaction, event quality, event expectations, performance, and memorable experiences and total expenditure without accommodation but none of the factors listed (with their corresponding items) have statistically significant coefficients, as indicated by their high *p*-values (all above 0.05).

**Figure 2:**
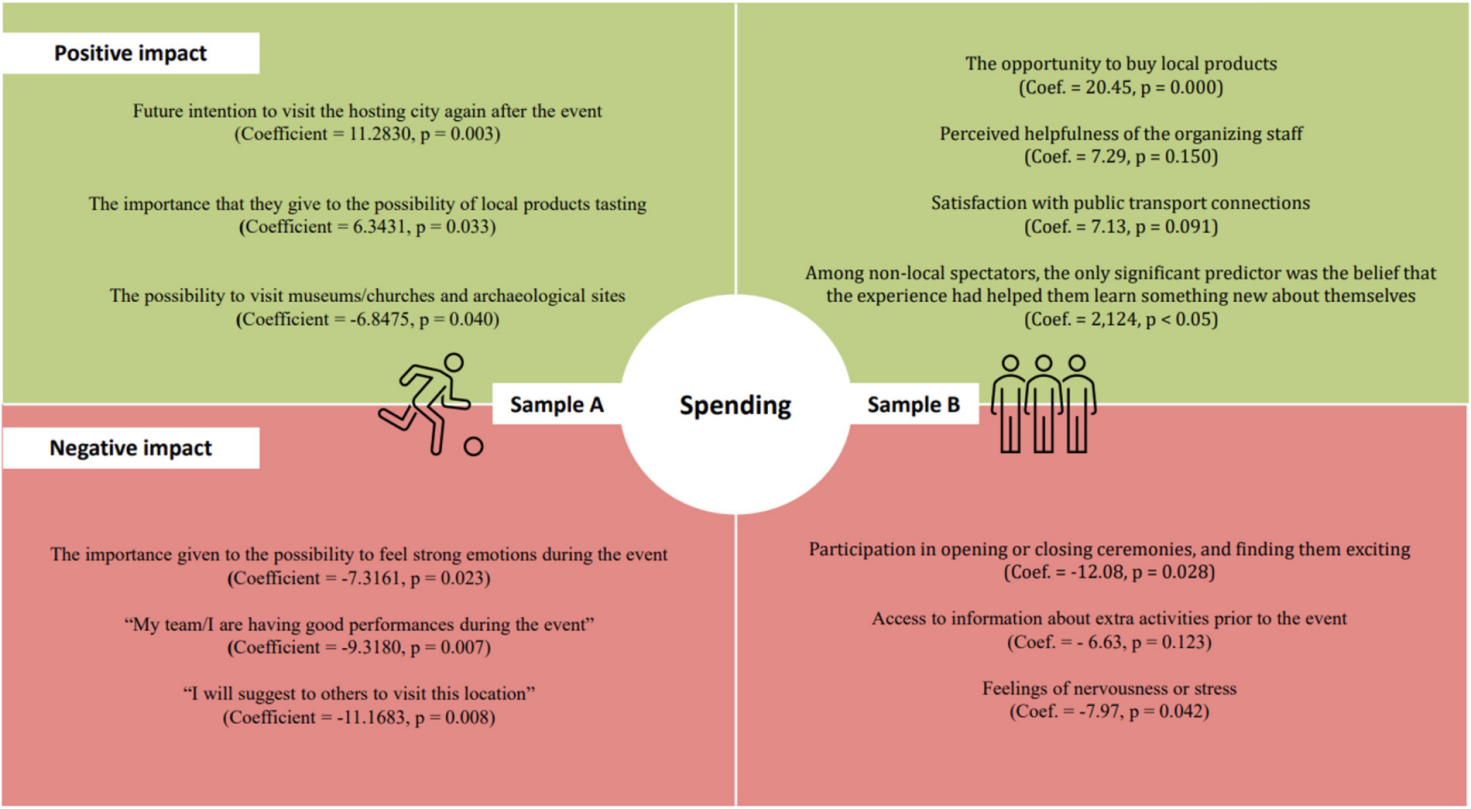
Main results of the regression analyses.

Figure 2 summarizes and compares the main results of the regression analyses of samples A and B, both having as a dependent variable the individual expenditure during the event. For Sample A, positive predictors included the intention to revisit the host city (*β* = 1.228, *p* < 0.01) and appreciation for local product experiences (*β* = 0.847, *p* < 0.05), while negative predictors included strong emotional reactions (*β* = −2.794, *p* < 0.01) and focus on team performance (*β* = −1.160, *p* < 0.05). For Sample B, spending was positively influenced by the opportunity to buy local products (*β* = 2.848, *p* < 0.01), satisfaction with staff (*β* = 7.286, *p* < 0.05), and perceived helpfulness of transportation services. Ceremony attendance and early access to information were negatively associated with spending.

From the data analyzed in the regression, where spending (accommodation excluded) was the dependent variable, we found that the overall model is not statistically significant at a typical level (e.g., 0.05), meaning the model as a whole does not significantly explain the variability in spending. Looking at the coefficients and *p*-values for individual variables, the analysis shows few significant variables with a positive impact on spending of athletes and staff. One of these variables is the importance that they give to the possibility of local products tasting (Coefficient = 6.3431, *p* = 0.033). Future intention to visit the hosting city again after the event is also positively associated to spending (Coefficient = 11.2830, *p* = 0.003). On the other hand, the importance given to the possibility to feel strong emotions during the event seems to have negative impact on spending (Coefficient = −7.3161, *p* = 0.023) as well as the possibility to visit museums/churches and archaeological sites (Coefficient = −6.8475, *p* = 0.040). Other variables having negative influence on spending are “my team/I are having good performances during the event” (Coefficient = −9.3180, *p* = 0.007), and “I will suggest to others to visit this location” (Coefficient = −11.1683, *p* = 0.008). While some variables are statistically significant, as already mentioned, the overall model does not have strong explanatory power, as indicated by the low R-squared (0.2152: About 21.52% of the variance in spending is explained by the model) and adjusted R-squared values (0.0190) (data not shown).

For the spectators' sample, the analysis revealed several key daily-spending patterns. Men, on average, tend to spend more than women (€19.3 vs. €16.9). Additionally, individuals over the age of 40, those earning between €28,000 and €55,000, and those with higher levels of education (with the exception of PhDs) tend to spend more. Stepwise regression analysis was used to explore the factors influencing spending, excluding accommodation, and identified several significant predictors (*p*-value <0.05) related to event and participant experiences, such as the importance of opening/closing ceremonies, staff helpfulness, additional activities, nervousness, local product tasting, and public transportation connections. The model explained approximately 17.53% of the variance in spending (R-squared = 0.1753), indicating a moderate level of explanatory power. While this isn't a very high R-squared, it suggests that other factors not included in the model may also play a role in explaining the dependent variable. The model is statistically significant overall, F (6, 120) = 4.25, Prob > F = 0.0006 with an adjusted R-squared of 0.1340, meaning that at least one of the independent variables is significantly associated with the dependent variable (spending excluding accommodation).

Several key findings emerged from the detailed spectator's regression analysis shown in Table 1. Participation in opening or closing ceremonies, and finding them exciting, was associated with a significant negative effect on spending (Coef. = −12.08, *p* = 0.028), suggesting that attendees may allocate their resources differently, reducing expenditures on other activities. Perceived helpfulness of the organizing staff positively influenced spending (Coef. = 7.29, *p* = 0.150), with higher levels of customer service correlating with increased spending, even if not statistically significant (*p* > 0.05).

**Table 1:** Regression analysis spectators’ spending (accommodation excluded).

Predictors	Coefficient	Std. Err.	*t*	*p* > |*t*|	95% conf. interval
Ceremonies	−12.08	5.44	−2.22	0.028	−22.85, −1.32
Helpful staff	7.29	5.03	1.45	0.150	−2.67, 17.26
Additional activities	−6.63	4.27	−1.55	0.123	−15.07, 1.82
Feeling nervous	−7.97	3.87	−2.06	0.042	−15.63, −0.31
Local food and beverage	20.45	4.62	4.42	0.000	11.30, 29.60
Public transportations	7.13	4.19	1.70	0.001	−1.16, 15.43
_cons	−1.92	22.50	−0.09	0.932	−46.47, 42.63

Access to information about extra activities prior to the event was negatively related to spending (Coef. = −6.63, *p* = 0.123), implying that participants who focus on such activities tend to reduce their expenditures elsewhere. Similarly, feelings of nervousness or stress were associated with lower spending (Coef. = −7.97, *p* = 0.042), suggesting that more nervous participants engage less in event-related spending. For each unit increase in nervousness, expenditures decrease by about 7.97 units. On the other hand, the opportunity to buy local products had the strongest positive effect on spending (Coef. = 20.45, *p* = 0.000), suggesting that people more interested in local products tasting are likely to spend more on other activities (e.g., local food and experiences). For each unit increase in the importance of local product tasting, spending excluding accommodation increase by 20.45 units. Satisfaction with public transport connections also positively impacted spending, though the evidence isn't strong (Coef. = 7.13, *p* = 0.091), highlighting the importance of accessibility in encouraging higher expenditures. Among non-local spectators, the only significant predictor was the belief that the experience had helped them learn something new about themselves, which was associated with a considerable increase in spending (Coef. = 2,124, *p* < 0.05). This suggests that non-local attendees who found the event personally enriching tend to spend more.

## 4 Discussion

The research suggests that medium-sized sporting events, such as the one under investigation, can have a significant impact on the local economy via direct expenditures in various sectors. The thorough examination of expenditures emphasizes the importance of these events in fostering employment opportunities and local businesses. The factors and trends that influence expenditure in our study are consistent with the results of previous research. Our investigation revealed substantial economic consequences, with a direct economic impact of €1,323,572. These results demonstrate the significant impact that medium-sized sporting events have on the local economy.

A comparison with prior research underscores multiple consistent elements that affect expenditure. For instance, some research (46–48) emphasizes the significance of socio-demographic variables, such as origin, income, and travel characteristics. These are in line with our findings, which show that non-local attendees, particularly those on a business trip, were the main drivers of the host economy, with an average daily expenditure of €81 on non-accommodation items. This result corresponds with the dominant trend of heightened spending by non-residents.

Moreover, the scientific literature indicates that gender and age may affect spending patterns. Some research (49) suggests males spend more than women at local sports events, while other studies, like ours, reveal no substantial gender disparity in overall expenditures (23, 47). Our findings described in Figure 1, indicated that spectators exhibited greater expenditure than athletes, supporting previous research (50) illustrating that spectators frequently contribute more to the local economy than other participants and that the expenditure of athletes participants is often limited in these events due to factors such as the short duration of stay, group arrangements, all-inclusive packages offered by the organizers to the participating teams/clubs and limited discretionary spending. Our findings align with prior evaluations conducted by UK Sport and the Sport Industry Research Centre (SIRC) implemented through the EventImpacts framework demonstrating that junior and sub-elite athlete-focused events generate relatively low daily expenditures (9). The CNU event that we analyzed seems to follow this pattern.

This study seeks to examine also the impact that event satisfaction, perceived quality, and memorable experiences have on spending behaviors. Some studies (23, 51) indicate that higher satisfaction and positive experiences are associated with greater expenditures. In our study none of the factors investigated, such as individual satisfaction, event quality, event expectations, performance, and memorable experiences, demonstrated a statistically significant effect on total spending without accommodation. This indicates that additional unmeasured variables may be affecting spending behavior, or that the relationships are more intricate than represented by this model.

Our findings reveal some notable patterns regarding the expenditure of athletes, staff, and spectators. Our results align with Solberg's findings that different visitor categories show differences in spending behavior depending on factors like purpose of visit, duration of stay, and who bears the cost (26). Our data similarly show lower expenditures by athletes (often subsidized or bundled) compared to spectators, supporting the argument that market segmentation is critical to accurate impact assessment.

For athletes and staff, as shown in Figure 2, spending was positively correlated with an interest in tasting local products and inclinations to return to the host city after the event. Nevertheless, the emotional investment of participants in the event, particularly those who were experiencing strong negative emotions or visiting local cultural locations such as museums or archaeological sites, had a negative impact on spending. These findings indicate that athletes and staff who are intensely preoccupied with the sporting aspect or tourist attractions may not priorities other forms of expenditure. Research suggests that emotions play a significant role in spectator behavior at sporting events. Positive emotions from a home team victory can increase purchase intentions, while negative emotions may lead to decreased spending (52).

Spectators also demonstrated varying spending patterns. Men, older adults, persons with mid-range incomes, and those possessing better educational qualifications (excluding PhDs) exhibited greater expenditure. Our results align with those of Barquet et al. (53), demonstrating that attendees from elevated socio-economic strata generally spend more, signifying a direct correlation between income levels and economic impact. The investigation revealed that for spectators, the opportunity to purchase local products, the availability of attentive staff, and knowledge regarding public transport connections were the most significant positive factors influencing participant expenditure. Daniels and Norman (54) discovered that visitors who perceived elevated levels of customer service were more inclined to spend, corroborating the positive correlation between supportive personnel and expenditure in our research. Conversely, going to exciting ceremonies, having the potential to participate in marginal activities, and feeling anxious were linked to lower spending levels. There is a well-established correlation between the influence of overall sentiment and purchasing behavior, as evidenced by a variety of studies that investigate the general impact of affective factors, including moods, emotions, and evaluations (55–58). Adverse emotions can markedly diminish customers' spending patterns and their propensity to engage in hospitality and tourist activities (59). Moreover, the adverse effect of involvement in opening or closing ceremonies on expenditure corresponds with the observations of Grix and Lee (60), who indicated that participants in ceremonial events may curtail spending on alternative activities due to the time and money allocated to these occasions. In line with common event dynamics, it is not unexpected that participants who attended the opening and closing ceremonies reported lower overall spending. These ceremonies often provide free catering or hospitality and require significant time commitment, which can limit opportunities for discretionary spending elsewhere. Consequently, some expenditure may shift from individual visitors to the event organizers' budget.

Attendees who perceived the event as personally enriching, especially those from outside the area, exhibited higher expenditure, underscoring the significance of crafting memorable and powerful experiences. Gibson et al. (61) conducted a study on event tourism that underscored the economic impact of non-local visitors, who generally spend more than local spectators, a conclusion corroborated by the current analysis.

These findings not only reflect the practical spending behavior of event visitors, but also support theoretical models suggesting that time allocation, visitor role, and bundled services influence economic outcomes especially in smaller-scale, non-spectator-driven events.

The main contribution of this paper is its integrative approach, which blends spending data with experiential variables such as satisfaction, perceived quality, emotional engagement, and behavioral intention. Our findings imply that psychosocial factors could mediate spending behavior, particularly in smaller-scale, athlete-centric events, whereas prior research frequently treats economic impact analysis as a purely financial evaluation.

This multi-layered approach contributes to knowledge in two ways: first, it underlines the importance of incorporating emotional and experiential factors into studies of the economic impact of medium-sized events; second, it expands the academic usefulness of instruments such as the DEC by showing how they can be incorporated into theoretical frameworks pertaining to event tourism, consumer behavior, and experiential economics.

### 4.1 Limitations and strengths

This research possesses several limitations. The sample is confined to a particular event, the 2022 Italian National University Games, which may not accurately represent other sporting events or areas. The regression models, although finding significant predictors of spending, accounted for just a little fraction of the variance in spending behavior, indicating that additional unmeasured factors may be affecting expenditure. This may reflect the complex and context-dependent nature of spending behavior at events where many costs are standardized or externally covered (e.g., by universities or organizers). Furthermore, personal characteristics may influence the qualitative experience of the event more than actual spending, especially in non-commercial contexts. Future studies could address this by expanding sample sizes, refining spending categories, or applying more advanced modeling techniques to explore interaction effects. Additionally, recall bias may impact self-reported spending data, which may have been under- or overestimated by participants. Although surveys were conducted during or immediately after the event to reduce these biases, inaccuracies may still have occurred. Future research could address this limitation through triangulation with external data sources such as local economic indicators, business revenue records, or electronic transaction data, which would allow for more accurate and robust impact assessment. Another limitation concerns the study's use of only a subset of items from specific validated scales rather than the entire instruments. Since some items, like those pertaining to brand loyalty or merchandise purchases, were irrelevant to the event type or participant profile, this decision was made to guarantee contextual relevance. While the internal consistency of the adapted scales was confirmed through Cronbach's alpha, no exploratory factor analysis was conducted to examine the dimensional structure of the constructs. This may have limited the breadth and comparability of the constructs measured. Moreover, despite being gathered, information on spectators' physical activity and other background factors for both athletes and spectators was not examined in this study. Future studies could incorporate these data to enable a more thorough assessment of the event's legacy and social impact.

Lastly, although the questionnaire asked respondents to report only spending directly related to the event, and accommodation data was used to approximate non-local visitors, some local spending may still have been included, which could have resulted in a slight overestimation of the net economic impact and in the possible inclusion of deadweight expenditure.

Nevertheless, the study has numerous advantages. The substantial sample size (*n* = 963) and categorization into separate groups (athletes/staff and spectators) facilitated comprehensive subgroup analysis. The application of bivariate and regression analyses facilitated a thorough comprehension of spending patterns and their drivers. Furthermore, the emphasis on both economic and experiential elements offers a comprehensive perspective on the event's impact.

### 4.2 Practical implications and future research

This research provides valuable insights for policymakers, local businesses, and event organizers who are seeking to increase the economic impact of medium-sized sporting events. A better customer service, a more efficient public transport, and the promotion of local products are key factors to improve visitor experiences. Given that satisfaction and event quality affect spending, organizers ought to prioritize the creation of memorable experiences, particularly for non-local attendees, to stimulate increased spending. Facilitating accessible information regarding local activities and maintaining favorable tourist interactions might enhance spending and encourage returning visitors. In particular, the correlation between spending and the availability of local products suggests that organizers could increase the economic impact of similar events by promoting greater exposure to local food, handicrafts, and cultural activities. Allowing regional vendors into the event and developing targeted promotional activities could support the local economy and could enhance the overall visitor experience. Moreover, event planners must judiciously equilibrate ceremonial functions with commercial prospects, since emotional engagement in these events may diminish spending on other products.

Future research may investigate the economic impacts of diverse sporting events across many areas and cultures, providing a comprehensive picture of spending patterns. Longitudinal studies could investigate the evolution of spending patterns over time, especially for repeating occurrences. Utilizing qualitative methods, such as interviews, may uncover the underlying motivations for participants' expenditures, while examining psychological elements like contentment and stress could improve our comprehension of their economic implications.

Future research could expand on this study in several ways. For example it could be interesting to determine how local context affects spending behavior by conducting comparative analyses of comparable events in other cities or regions. Using more advanced econometric techniques could also improve the precision of estimates for determinants of spending. Lastly, incorporating qualitative methods, such as interviews with local businesses, residents, or policymakers, could provide a richer understanding of the event's perceived value and social impact beyond economic figures.

### 4.3 Conclusions

This research provides useful insights, illustrating how impactful medium-sized sporting events, such as the CNU under investigation here, may be on local economies. According to the data, they might generate income from direct spending on accommodation, food, travel, and retail, with non-local attendees contributing more than athletes. Another insight is that spending is strongly influenced by several variables, such as socio-demographics, event satisfaction, and customer service, and can be further enhanced through the promotion of local attractions and the improvement of public transport connections. Conversely, emotional investment in ceremonies and events may result in lower spending elsewhere.

The study also shows that organizers should focus on enhancing accessibility, customer service, and local involvement opportunities to maximize economic benefits. While this study offers valuable insights, it also emphasizes the necessity of conducting future research that investigates a broader range of events and analyses the evolution of spending patterns over time. Although this study has limitations, it provides useful information about how to take advantage of medium-sized sporting events to promote community involvement and local economic growth.

## Data Availability

The raw data supporting the conclusions of this article will be made available by the authors, without undue reservation.
